# Oral health behind the bars: oral health seeking behavior among jail prisoners at central jail of Peshawar, Pakistan: a cross-sectional study

**DOI:** 10.1186/s12903-023-03705-5

**Published:** 2023-12-08

**Authors:** Zia Ul Haq, Kashif Nawaz, Shamsul Alam, Farhad Ali Khattak, Naeem Ullah, Ahmed, Sobia Anwar, Abid Rahim, Saima Afaq, Syed Nasir Shah

**Affiliations:** 1 Khyber Medical University, Peshawar, Pakistan; 2 Health Department, Khyber Pakhtunkhwa, Pakistan; 3 Khyber College of Dentistry, Peshawar, Pakistan; 4 Sardar Begum Dental College, Gandhara University, Peshawar, Pakistan; 5 Imperial College London, London, UK; 6 Saidu Medical College, Swat, Pakistan; 7 University of York, york, United Kingdom

**Keywords:** Dentist-patient relations. Oral health, Oral Hygiene, Prisoners, Health Behavior

## Abstract

**Background:**

The oral health care-seeking behavior among prison inmates is an overlooked area, often leading to deteriorating general health due to the prisoners’ limited awareness of oral hygiene practices. It is crucial to address this issue and understand the factors associated with oral healthcare-seeking behavior in prisons.

**Objective:**

To assess the oral healthcare-seeking behavior of prison inmates at Central Prisoner Jail, Peshawar Pakistan and to look the factors associated with their dental care utilization.

**Material and Methods:**

This cross-sectional study was conducted at Central Prisoner Jail, Peshawar Khyber Pakhtunkhwa, Pakistan from November 2021 to April 2022. A consecutive sampling technique was used to collect data from both convicted and under-trial prisoners by using a pre-tested WHO Basic Oral Health Survey 2013 tool. Our outcome variable was “Visit to a dentist in the last 12 months (Never/Once or more than one visit). Chi-square test was used to determine univariate association with other explanatory variables while multivariable logistic regression was performed to adjust for potential confounders.

**Result:**

A total of 225 participants were recruited to the study with a mean (SD) age of 32.9(11.4). More than two-thirds of 200(88.9%) of the participants were males. One-third of the sample never visited the dentist75(33.3). Participants who completed college/university education and never visited the dentist in the last 12 months constituted a smaller proportion (17.6%) compared to those who visited the dentist once or more than once *n* = 28(82.4%, *p*-value = 0.003). Individuals who were using toothbrushes were most frequently visiting the dentist *n* = 130(72.6%=*p* value = 0.001) as compared to never visitors. Multivariate logistic regression analysis revealed that Participants who experienced teeth pain or discomfort had 0.42 times lower odds of visiting the dentist compared to those who did not experience any pain or discomfort [AOR 0.42 (95% CI 0.17–0.80), *p* = 0.04]. Similarly, Participants who do not use any denture have 4.06 times higher odds[AOR 4.06(95% CI 1.76–9.36), *p* = 0.001] of visiting the dentist compared to those who use a denture.

**Conclusion:**

Our result demonstrates that those prisoners who were experiencing tooth pain or discomfort and not using dentures were the strong predictors with lower dental visit frequency to seek oral health care.

**Supplementary Information:**

The online version contains supplementary material available at 10.1186/s12903-023-03705-5.

## Background

Every citizen has been considered to have a fundamental human right to a quality health service while oral health is an integral part of our general health [1]. Dental health is the main indicator of a better quality of life, wellness, and overall health [2]. Prisoners are a marginalized and unique population having numerous health including poor oral health [3]. The quality of life of jail inmates is significantly impacted by oral issues, which can also affect their ability to eat and speak and cause pain and discomfort [4]. Despite the high prevalence of oral health problems among Pakistani prisoners, little is known about their dental care-seeking behavior and access to health care services [5] which highlights the need for improvement in the primary health care system [6].

According to the world prison brief, there are about 87,000 prisoners in Pakistan this number has increased by over 50% in the last decade [7]. The majority of the prisoner come from disadvantaged socioeconomic backgrounds and their oral health is often neglected [8]. Lack of access to dental care is a significant problem for inmates in Pakistani prisons and little is known about the dental care-seeking behavior of this population [9, 10].

There is a substantial literature gap in understanding the oral health care needs and utilization patterns of prisoners in low- and middle-income countries [11]. In India, the study conducted by Puneet Kumar et al. stated that the quality and quantity of dental health care was below the acceptable limit like 98.2% of inmates has incidence of tooth decay. In another study carried out in Karachi, Pakistan, researchers delved into the relationship between tobacco consumption and its effects on dental health, with a particular emphasis on incarcerated women in a prison environment. The results brought to the forefront a significant link between substance use and an increased severity of periodontitis, spotlighting the worrisome health status among this specific demographic of incarcerated females [12]. But on the other hand, most of the existing literature on this topic has focused on high-income countries where prisoners have better access to dental care and general health [13].In the United States, for example, the rate of tuberculosis among prisoners is nearly 100 times higher than that of the general population, while the rate of HIV infection is more than three times higher than normal population [14]. Similarly, a study conducted in Canada found that prisoners had a higher prevalence of dental disease and were less likely to seek dental care compared to the general population [15].

Studies have shown that prisoners have significantly worse oral health than the general population, which may be attributed to a lack of access to dental care and the stressful prison environment, which includes social isolation from society and family. Recognizing the significance of healthcare-seeking behavior (HCB) as the actions individuals take when they perceive a health issue or illness, it becomes crucial to comprehend these behaviors and the underlying factors. This understanding empowers governments to effectively allocate and manage available healthcare resources, ultimately ensuring the well-being of the population. So this study will be the first one in the region that aims to assess the oral healthcare-seeking behavior of prison inmates at Central Prisoner Jail, Peshawar Pakistan and to look the critical factors influencing their approach to dental care utilization.

## Materials and methods

### Study setting and sample size

This cross-sectional study was done from November 2021 to April 2022 on prison inmates of Central Prison, Peshawar- Pakistan which is the largest provisional jail in Khyber Pakhtunkhwa Pakistan [16]. By using the WHO sample size calculator, the total required sample size was 225, using 82% frequency of prison inmates’ respondents about having not regular dental visitors while taking a 5% margin error and 95% confidence interval [17].

### Ethical clearance

Ethical clearance for this study was acquired from the University’s institutional ethical council vide notification No DIR/KMU-EB/DC/000105. In addition, the Inspector General of Police, and the Inspector General of Prisons, KPK, gave their approval for data collection. Informed written consent was also acquired from those prison inmates who were willing to participate in this study. For jail inmates below the age of 16 years, written informed consent was obtained from their parents/guardians. In addition to this, all the methods/procedures were performed in accordance with the relevant guidelines and regulations (Declaration of Helsinki).

### Data collection procedure

For the commencement of this study, formal permission was solicited from the Inspector General of Prisoner. We included all the convicted and under-trial prisoners while excluding inmates with crippled health and those prisoners who were in terrorist barracks. A pre-tested World Health Organization (WHO) Basic Oral Health Survey 2013 questionnaire with a Cronbach’s alpha of 0.88, indicating high internal consistency [18] was employed to assess the oral health behavior of participants. Additionally, demographic information such as age and gender was collected. The questionnaire also included inquiries regarding reasons for seeking dental healthcare and problems arising from inadequate oral and dental hygiene practices [19] was used to determine the oral health behavior of the participants, accompanied by demographic particulars such as age, gender; questions associated with the reason for visiting dental health care providers, and problems arising due to poor oral and dental hygiene were included. The study was conducted in the Dental Section of Central Prison Hospital Peshawar. The study participants were accommodated on a dental chair at an estimated space of six feet following the standard operating procedures of the COVID-19 pandemic. Different dental instruments were used during this study for the examination of inmates like a mouth mirror, probe, surgical glove, mask, torch, and face shield. After conducting this study, the participants were advised regular dental checkups by a Dentist, some of them were medicated according to their needs while some of them were treated accordingly as per routine checkups.

### Data analysis

Data were analyzed through SPSS version 22. All the categorical variables were presented in the form of frequency and percentages while continuous variables were presented in the form of mean and standard deviation and the n were converted to categorical variables by dividing the range into 3. Taking a visit to a dentist in the last 12 months (Never/Once or more than one time) was taken as an outcome variable and tested for association with all other independent variables by using the chi-square test. Similarly, logistic regression was used to look for predictors while multi-logistic regression was applied to adjust for confounders. *P*-value ≤ 0.05 was taken as statistical significance.

## Results

### Descriptive statistics of socio-demographic characteristics

A total of 225 participants including convicted and under-trial adult prisoners were recruited for the study. The mean age of the participants was 32.9 (SD 11.4), of which 200 (88.9%) were males. About a quarter of the participants 54 (24.0%) completed secondary school education and an almost equal number of participants were from Urban, per-urban areas of Peshawar, Pakistan.

### Oral health and dental care-seeking behavior practices of the participants

Our result demonstrates that one-third of the participants never visited the dentist in the last 12 months 75(33.3) as compared to those who visited one or more than one time 150(66.7%). The proportion of individuals who had not visited the dentist in the last 12 months and had completed college/University education was lower 06(17.6%) compared to the proportion of individuals who had visited the dentist once or more *n* = 28(82.4%=*p* value = 0.003). Participants who lived in urban areas visited the dentist once a day or more were more than three quarters *n* = 54(73.0%, *p* value = 0.36). Those having pain in their teeth or mouth caused pain and discomfort during the past 12 months observed more frequent visits to the dentist once a day or more is *n* = 90(59.2%, *p* value = 0.001) as compared to those who never visit. More than half *n* = 38(60.3%, *p* = 0.001), who were partial denture users, never visit a dentist who had a partial denture (Table 1).

Table 2 demonstrates the result of the items used to clean the teeth by the jail inmates. Individuals who cleaned their teeth with toothbrushes visited the dentist more than half of the participants were *n* = 130(72.6%, *p*-value = 0.001). Participants who don’t use toothpicks to clean their teeth visit the dentist are almost more than half *n* = 112(63.3%, *p* value = 0.03). Pain or trouble in teeth, gums, or mouth was the most common reason to visit the dentist *n* = 85(62.5%, *p* value0.05). Of those using toothpaste for oral hygiene (129, 72.9%), a significant majority visited the dentist frequently, whereas only 48 (27.1%) who never visited the dentist demonstrated this behavior (*P* value = 0.001).

Table 3 shows the problems during the past 12 months. Those participants who don’t feel embraced due to the appearance of their teeth visited the dentist once a day or more were frequently in number *n* = 123(68.7%, *p*-value = 0.75). participants who do not avoid smiling during the past 12 months due to teeth appearance visited the dentist more in number *n* = 129(69.7%, *p* value = 0.06). Individuals who reported frequent dental visits and uninterrupted sleep numbered 108 (74.0%), in contrast to 38 (26.0%) who never visited the dentist (*P* value = 0.002). Table 4 shows the dietary habits of the participants before prison. Looking at the pattern of sweet consumption, 33 individuals (41.3%) reported consuming sweets seldom or never, and among them, the majority never visited their doctor. On the other hand, more than half of the participants *n* = 47(58.8%,*p* value = 0.03) reported frequent visits to their dentists. Individuals using snuff several times a day demonstrated a higher frequency of dental visits, with 22 (55.0%) never visiting the dentist, compared to 18 (45.0%) who visited once or more than once.

**Table 1:** Association of dependent variable with different explanatory variables

Variable	Categories	How often do you visit to the dentist in the last 12 months?		*P* value
		Never	Once or more than one time	
Gender	Male	68(34.0%)	132(66.0%)	0.54
	Female	7(28.0%)	18(72.0%)	
Age in years	13–34	34(34.8%)	90(85.2)	0.688
	35–53	24(32.4%)	50(67.5%)	
	More than 53	03(23.1%)	10(76.9%)	
Level of education	No formal schooling	7(50.0%)	7(50.0%)	0.003
	Less than Primary School	15(46.9%)	17(53.1%)	
	Primary school completed	24(50.0%)	24(50.0%)	
	Secondary school completed	13(24.1%)	41(75.9%)	
	High School completed	10(23.3%)	33(76.7%)	
	College/University completed	6(17.6%)	28(82.4%)	
Location	Urban	20(27.0%)	54(73.0%0)	0.36
	Peri urban	26(35.6%0)	47(64.4%)	
	Rural	29(37.2%0)	49(62.8%)	
How many natural teeth you have?	1–9 Teeth	1(50.0%)	1(50.0%)	0.08
	10–19 Teeth	16(50.0%)	16(50.0%)	
	20 teeth or more	58(30.4%)	133(69.6%)	
During the past 12 months, did your teeth or mouth cause any pain or discomfort?	No	13(17.8%)	60(82.2%)	0.001
	Yes	62(40.8%)	90(59.2%)	
Do you use any removable denture?	A partial denture	38(60.3%)	25(39.7%)	0.001
	A full upper denture	4(30.8%)	9(69.2%)	
	A full lower denture	4(50.0%)	4950.0%)	
	No	29(20.6%)	112(79.4%)	
How would you describe the state of your teeth and gums?	Excellent	0(0.0%)	5(100.0%)	0.001
	Very good	15(26.3%)	42(73.7%)	
	Good	16(21.1%)	60(78.9%)	
	Average	34(45.9%)	40(54.1%)	
	Poor	10(76.9%)	3(23.1%)	
How often do you clean your teeth?	Never	1(100.0%)	0(0.0%)	0.001
	Once a month	12(100.0%)	0(0.0%)	
	Once a week	62(100.0%)	0(0.0%)	
	Once a day	0(0.0%)	130(100.0%)	
	Twice or more a day	0(0.0%)	20(100.0%)	

Chi-square test | *p* ≤ 0.05 = statistically significant | *p* = 0.001 = highly significant

**Table 2:** Do you use any of the following items to clean your teeth?

Variable	Categories	How often do you visit the dentist in the last 12 months??		*P* value
		Never	Once or more than one time	
Toothbrush	No	26(56.5%)	20(43.5%)	0.001
	Yes	49(27.4%)	130(72.6%)	
Toothpicks	No	65(36.7%	112(63.3%	0.03
	Yes	10(20.8%	38(79.2%	
Dental floss	No	74(34.3%)	142(65.7%)	0.14
	Yes	01(11.1%)	8(88.9%)	
Miswak	No	24(27.9%)	62(72.1%)	0.17
	Yes	51(36.7%)	88(63.3%)	
Other	No	75(33.8%)	147(66.2%)	0.21
	Yes	0(0.0%)	3(100.0%)	
Do you use toothpaste to clean your teeth?	No	27(56.3%)	21(43.8%)	0.001
	Yes	48(27.1%)	129(72.9%)	
Do you use toothpaste that contains fluoride?	No	73(40.8%)	106(59.2%)	0.001
	Yes	2(4.3%)	44(95.7%)	
What was the reason for your last visit to the dentist?	Consultation/advise	6(22.2%)	21(77.8%)	0.05
	Pain or trouble with teeth, gums, or mouth	51(37.5%)	85(62.5%)	
	Follow-up	18(34.6%)	34(65.4%)	
	Routine check-up/treatment	0(0.0%)	10(100.0%)	

Chi-square test | *p* ≤ 0.05 = statistically significant | *p* = 0.001 = highly significant

**Table 3:** Experienced any of the problems during the past 12 months before prison?

Variable	Categories	Visit to the dentist in the last 12 months?		*P* value
		Never	Once or more than one time	
Difficulty in biting food	Don’t Know	1(33.3%)	2(66.7%)	0.001
	No	45(26.5%)	125(73.5%)	
	Sometimes	25(58.1%)	18(41.9%)	
	Fairly Often	4(57.1%)	3(42.9%)	
	Very Often	0(0.0%)	2(100.0%)	
Difficulty in Chewing Food	Don’t Know	29(25.4%)	85(74.6%)	0.001
	No	36(37.5%)	60(62.5%)	
	Sometimes	10(83.3%)	2(16.7%)	
	Fairly Often	0(0.0%)	2(100.0%)	
	Very Often	0(0.0%)	1(100.0%)	
Difficulty in Speech trouble	Don’t Know	2(50.0%)	2(50.0%)	0.006
	No	60(30.0%)	140(70.0%)	
Dry mouth	Sometimes	11(73.3%)	4(26.7%)	0.002
	Fairly Often	2(33.3%)	4(66.7%)	
	Don’t Know	0(0.0%)	1(100.0%)	
	No	29(34.1%)	56(65.9%)	
Felt embarrassed about the appearance of Teeth	Sometimes Fairly Often	17(60.7%) 26(31.7%)	11(39.3%) 56(68.3%)	0.75
	Very Often	3(10.3%)	26(89.7%)	
	Don’t Know	2(33.3%)	4(66.7%)	
	No	56(31.3%)	123(68.7%)	
	Sometimes	8(42.1%)	11(57.9%)	
	Fairly Often	7(43.8%)	9(56.3%)	
	Very Often	2(40.0%)	3(60.0%)	
Felt stressed due to oral or dental problems	Don’t Know	4(100.0%)	0(0.0%)	0.005
	No	48(28.1%)	123(71.9%)	
	Sometimes	11(52.4%)	10(47.6%)	
	Fairly Often	7(46.7%)	8(53.3%)	
	Very Often	5(35.7%)	9(64.3%)	
Avoided smiling due to problems in teeth	Don’t Know	2(50.0%)	2(50.0%)	0.06
	No	56(30.3%)	129(69.7%)	
	Sometimes	9(64.3%)	5(35.7%)	
	Fairly Often	5(50.0%)	5(50.0%)	
	Very Often	3(25.0%)	9(75.0%)	
Sleep often interrupted	Don’t Know	38(26.0%)	108(74.0%)	0.002
	No	27(56.3%)	21(43.8%)	
	Sometimes	3(33.3%)	6(66.7%)	
	Fairly Often	7(31.8%)	15(68.2%)	
	Very Often	38(26.0%)	108(74.0%)	
Have taken days off from work?	Don’t Know	50(36.8%)	86(63.2%)	0.49
	No	5(31.3%)	11(68.8%)	
	Sometimes	11(24.4%)	34(75.6%)	
	Fairly Often	9(32.1%)	19(67.9%)	
	Very Often	50(36.8%)	86(63.2%)	
Difficulty in doing usual activities	Don’t Know	3(60.0%)	2(40.0%)	0.004
	No	58(30.2%)	134(69.8%)	
	Some-times	12(70.6%)	5(29.4%)	
	Fairly Often	0(0.0%)	4(100.0%)	
	Very Often	2(28.6%)	5(71.4%)	
Felt Less tolerant to people like spouse/close people	Don’t Know	13(50.0%)	13(50.0%)	0.17
	No	57(31.7%)	123(68.3%)	
	Some-times	4(40.0%)	6(60.0%)	
	Fairly Often	1(25.0%)	3(75.0%)	
	Very Often	0(0.0%)	5(100.0%)	
Have reduced participation in social activities	Don’t Know	2(66.7%)	1(33.3%)	0.28
	No	70(33.3%)	140(66.7%)	
	Some-times	1(12.5%)	7(87.5%)	
	Fairly Often	1(33.3%)	2(66.7%)	
	Very Often	1(100.0%)	0(0.0%)	

Chi-square test | *p* ≤ 0.05 = statistically significant | *p* = 0.001 = highly significant

**Table 4:** How often do you eat or drink any food before prison?

Variable	Categories	Visit to the dentist in the last 12 months?		*P* value
		Never	Once or more than one time	
Fresh fruit	Never	7(70.0%)	3(30.0%)	0.02
	Several times a month	51(36.4%)	89(63.6%)	
	Once a week	2(15.4%)	11(84.6%)	
	Several times a week	6(16.7%)	30(83.3%)	
	Every week	6(31.6%)	13(68.4%)	
	Several times a day	3(42.9%)	4(57.1%)	
Biscuits	Seldom/never	16(38.1%)	26(61.9%)	0.03
	Several times a month	34(35.1%)	63(64.9%)	
	Once a week	5(83.3%)	1(16.7%)	
	Several times a week	14(25.9%)	40(74.1%)	
	Every week	3(15.0%)	17(85.0%)	
	Several times a day	3(50.0%)	3(50.0%)	
Sweets	Seldom/never	33(41.3%)	47(58.8%)	0.03
	Several times a month	11(20.0%)	44(80.0%)	
	Once a week	9(31.0%)	20(69.0%)	
	Several times a week	17(47.2%)	19(52.8%)	
	Every week	5(20.8%)	19(79.2%)	
	Several times a day	0(0.0%)	1(100.0%)	
Jam/honey	Seldom/never	48(40.0%)	72(60.0%)	0.12
	Several times a month	8(19.5%)	33(80.5%)	
	Once a week	3(27.3%)	8(72.7%)	
	Several times a week	12(40.0%)	18(60.0%)	
	Every week	4(21.1%)	15(78.9%)	
	Several times a day	0(0.0%)	3(100.0%)	
Sweet/candy	Seldom/never	46(36.8%)	79(63.2%)	0.21
	Several times a month	7(18.9%)	30(81.1%)	
	Once a week	5(45.5%)	6(54.5%)	
	Several times a week	9(45.0%)	11(55.0%)	
	Every week	3(27.3%)	8(72.7%)	
	Several times a day	5(23.8%)	16(76.2%)	
Tea with sugar	Seldom/never	1(33.3%)	2(66.7%)	0.17
	Several times a month	0(0.0%)	4(100.0%)	
	Once a week	9(23.1%)	30(76.9%)	
	Several times a week	1(100.0%)	0(0.0%)	
	Every week	64(36.0%)	114(64.0%)	
	Several times a day	1(33.3%)	2(66.7%)	
Cigarette	Seldom/never	31(36.0%)	55(64.0%)	0.21
	Several times a month	2(66.7%)	1(33.3%)	
	Several times a week	7(20.0%)	28(80.0%)	
	Every week	0(0.0%)	2(100.0%)	
	Several times a day	35(35.4%)	64(64.6%)	
Use of snuff	Seldom/never	48(28.7%)	119(71.3%)	0.02
	Several times a month	1(33.3%)	2(66.7%)	
	Once a week	1(100.0%)	0(0.0%)	
	Several times a week	3(25.0%)	9(75.0%)	
	Every week	0(0.0%)	2(100.0%)	
	Several times a day	22(55.0%)	18(45.0%)	
Other tobacco products	Seldom/never	74(33.5%)	147(66.5%)	0.72
	Several times a day	1(25.0%)	3(75.0%)	

We accommodate all the independent variables with *p*-value ≤ 0.05 to the final regression model in Table 5. Visit to the dentist during the last 12 months “never” or “Once or more than one time” was taken as the dependent variable while those with a significant *P* value were selected as covariates.

After controlling all the factors, the results of multivariate logistic regression analysis found only two significant covariates to be associated with the dependent variable (visit to the dentist in the last 12 months). Table 5 showed that participants who experienced tooth pain or discomfort had 0.42 times lower odds (adjusted odds ratio) of visiting the dentist compared to those who did not experience any pain or discomfort (AOR 0.42, 95% CI 0.17–0.80, *p* = 0.04). Additionally, participants who did not use dentures had 4.06 times higher odds of visiting the dentist compared to those who used a denture (AOR 4.06, 95% CI 1.76–9.36, *p* = 0.001).

**Table 5:** Logistic regression analysis of participant’s characteristics associated with the dependent variable (visit to the dentist in the last 12 months)

Characteristic	Univariate analysis (Unadjusted OR)	*P* value	Multivariate analysis (Adjusted OR)	*P* value
**How many natural teeth do you have: Reference (1 to 9 teeth)**				
10–19 Teeth	1.00(0.06–17.41)	1.01	0.43(0.01–18.51)	0.66
20 teeth or more	2.29(0.141–37.29)	0.56	0.31(0.006–15.76)	0.56
**During the past 12 months did your teeth cause any pain or discomfort: Reference (no)**				
Yes	0.31(0.159–0.62)	0.001	0.42(0.17–0.80)	0.04
**Do you use any dentures? Reference (a partial denture)**				
A full upper denture	3.42(0.950–12.32)	0.06	4.11(0.71–23.96)	0.12
A full lower denture	1.520(0.348–6.64)	0.58	0.99(0.16–6.23)	0.99
No	5.870(3.067–11.24)	0.001	4.06(1.76–9.36)	0.001
**Do you use a toothbrush to clean your teeth? Reference (no)**				
Yes	3.45(1.77–6.73)	0.001	0.79(0.14–4.53)	0.79
**Do you use toothpicks to clean your teeth?: Reference (no)**				
Yes	2.20(1.03–4.72)	0.04	1.30(0.48–3.54)	1.30
**Do you use toothpaste to clean your teeth? Reference (no)**				
Yes	3.45(1.79–6.68)	0.001	3.32(0.66–16.80)	0.147
**Experiences any of the problems during the past 12 months**: Difficulty speech trouble: **Reference (Don’t know)**				
No	2.33(0.32–16.95)	0.40	6.31(0.56–70.91)	0.14
Sometimes	0.36(0.04–3.52)	0.38	2.81(0.16–47.94)	0.47
Fairly Often	2.00(0.15–26.73)	0.60	9.49(0.35-259.51)	0.18
**Experiences any of the problems during the past 12 months**: Avoid smiling because of teeth problems: **Reference (Don’t know)**				
No	2.30(0.32–16.8)	0.410	1.13(0.086–14.79)	0.92
Sometimes	0.56(0.06–5.24)	0.61	0.96(0.06–16.07)	0.98
Fairly Often	1.00(0.10-10.17)	1.00	0.59(0.03–12.10)	0.73
Very Often	3.00(0.29–31.63)	0.36	1.75(0.08–37.96)	0.72
**Experiences any of the problems during the past 12 months**: sleep often interrupted? **Reference (no)**				
Sometimes	0.27(0.139–0.54)	0.001	0.60(0.22–1.60)	0.30
Fairly Often	0.704(0.168–2.95)	0.63	1.57(0.24–10.42)	0.64
Very Often	0.754(0.286–1.99)	0.57	0.80(0.24–2.59)	0.71
**How often do you eat or drink any food**? fruit juice: **Reference (seldom/ never)**				
Several times a month	4.072(1.01–16.44)	0.05	1.82(0.24–13.96)	0.56
Once a week	12.83(1.69–97.19)	0.01	10.6(0.538-212.166)	0.12
Several times a week	11.67(2.33–58.46)	0.003	3.83(0.37–40.08)	0.26
Every week	5.06(0.96–26.66)	0.06	2.18(0.19–24.20)	0.53
Several times a day	3.11(0.41–23.39)	0.27	2.02(0.10-38.57)	0.64
**How often do you eat or drink any food?** biscuit: **Reference (seldom/ never)**				
Several times a month	1.14(0.54–2.41)	0.73	0.48(0.16–1.45)	0.19
Once a week	0.12(0.01–1.15)	0.07	0.06(0.003–1.35)	0.08
Several times a week	1.76(0.74–4.20)	0.20	0.62(0.18–2.12)	0.45
Every week	3.49(0.88–13.81)	0.07	0.99(0.16–6.22)	0.99
Several times a day	0.61(0.11–3.43)	0.58	0.43(0.04–5.05)	0.50
**What level of education have you completed?: Reference (no formal schooling)**				
Less than Primary School	1.13(0.32–3.98)	0.84	1.173(0.19–7.17)	0.86
Primary school completed	1.00(0.30–3.29)	1.00	0.663(0.12–3.72)	0.64
Secondary school completed	3.15(0.93–10.67)	0.06	1.773(0.333–9.44)	0.50
High School completed	3.30(0.93–11.68)	0.06	1.911(0.34–10.66)	0.46
College/University completed	4.67(1.19–18.35)	0.03	2.05(0.34–12.50)	0.43

## Discussion

In this cross-sectional study, we aimed to explore the dental care-seeking behavior of prison inmates at Central Prison, Peshawar, Pakistan, utilizing the WHO Basic Oral Health Survey 2013 tool. A total of 225 adult prisoners, comprising both convicted and under-trial individuals, were enrolled. Notably, participants residing in urban areas exhibited a higher frequency of dental visits, whereas more than half of those with partial dentures reported never visiting a dentist. The most prevalent motivation for dental visits was pain or discomfort in teeth, gums, or mouth. Participants with a tooth count between 10 and 19 were less inclined to seek dental care. Moreover, our study uncovered an intriguing trend: among participants who reported rare or no consumption of sweets, there was a notable preference for frequent dental visits (once or more than once). Additionally, inmates who employed a toothbrush for oral hygiene maintenance were the most regular visitors to the dentist, while those using toothpicks were also prominently represented among dental care seekers. Notably, individuals reporting no speech difficulties over the past 12 months exhibited a higher frequency of dental check-ups. Conversely, participants experiencing tooth pain or discomfort were less likely to seek dental care compared to their counterparts without such discomfort. Similarly, those who did not use dentures demonstrated a higher likelihood of visiting the dentist compared to denture users.

In our study, prisoners who used to live in urban areas visits dentists more frequently than those who lived in rural areas although the result were statistically non-significant. A study conducted in the US by Huabin Luo to assess rural-urban differences in dental service demonstrated that adults from rural areas were less likely to receive oral procedures [20]. These findings demonstrate that individuals living in urban areas have greater access to dental care and may be more aware of the importance of oral health. These findings highlight the need to improve access to dental care in rural areas where jails are located so to reduce oral health disparities.

Another important finding of this study was that more than half of the participants who had partial dentures never visited the dentist. A study in the UK with a multi-center approach showed that those with partial dentures feel embracement and stigma and avoid visiting the hospital or cleaning their denture [21]. Another study conducted in India aimed to assess the oral health status and treatment needs of jail prisoners. The results revealed that 8.8% of the study population had oral dentures.These results suggest that there may be a lack of education and awareness among the prisoner population about the importance of regular dental checkups, particularly for those with partial dentures who require specialized care.

Pain or discomfort in teeth, gums, or mouth was the most common reason cited by participants for visiting the dentist. A similar trend was also observed by Muhanad Alhareky in Saudi Arabia, where pain was the most common reason to visit the dentist [22]. Similarly, another study conducted in Saudi Arabia reported that 95.1% of Janil prisoners sought dental care due to tooth pain [23]. This research emphasizes the need to inform prisoner about the value of preventive care and routine dental examination, as delaying the treatment until the symptoms appear can result in expensive procedures.

Our study revealed that among participants who reported consuming sweets seldom or never, more than half (58.8%, *n* = 47) demonstrated a significant inclination towards frequent dental visits. This finding suggests that individuals who consume sweets less frequently are more likely to prioritize their dental health by seeking regular dental care [24].

A study conducted in Thailand among prisoners revealed that the majority of participants believed that increasing the frequency of tooth brushing and reducing their consumption of sugary beverages could help alleviate dental discomfort and ultimately lead to fewer dental visits [25]. Those with 10 to 19 teeth were less likely to visit the dentists once or more. These findings may relate to the fact that participants with fewer teeth may have less concern about maintaining their oral health or may have difficulty accessing dental care due to physical or financial barriers [26].

Finally, those who had no speech issues in the previous 12 months were more likely to go to the dentist. This research shows that those who have trouble speaking may find it difficult to get dental treatment or may not realize how important regular dental examinations are. A study conducted by Appukuttan D [27] stated that dental anxiety, phobia, and speech issues create huddles in the avoidance of dental care and suggested cognitive and psychotherapeutic interventions.

Finally, in our study, inmates who utilized a toothbrush for oral hygiene were the most consistent attendees of dental check-ups, and toothpick users were also notably represented among those seeking dental care. In contrast, a study conducted in Nigeria revealed that approximately 89.3% of prisoner participants had never heard of dental floss, and only a mere 1.8% had ever used it [28]. This discrepancy suggests a potential variation in oral hygiene practices and awareness between the two populations of jail prisoners.

While this study offers valuable insights into dental care-seeking behavior among prison inmates, it has certain limitations. Its cross-sectional design prevents causal conclusions, providing only a snapshot. Conducted in a single center, generalizability to broader inmate populations may be limited. Future research with a longitudinal, multi-center approach could provide a more comprehensive understanding.

## Conclusion

In conclusion, our study highlights significant factors influencing dental care-seeking behaviour among prison inmates. Oral pain emerged as strong determinants of frequent dental visits. Participants with partial dentures exhibited a noteworthy trend of non-attendance. Maintaining oral hygiene through toothbrush and toothpick usage correlated with higher dental visitation rates. Speech difficulties and tooth discomfort impacted visitation patterns. Importantly, those without dentures were more inclined to seek dental care. Addressing dental pain, advocating oral hygiene, and recognizing denture use are pivotal in enhancing healthcare-seeking behavior among incarcerated individuals.

### Implications of the study

This study offers crucial insights for oral healthcare in low and middle-income countries, particularly among incarcerated populations. Addressing barriers to dental access and awareness is imperative. Higher education correlates with greater dental care utilization, highlighting the need for tailored outreach to less-educated individuals. Emphasizing basic oral hygiene practices, such as toothbrush use, is pivotal in promoting dental visits. Proactive, preventive care measures are essential, given the inverse relationship between dental discomfort and visits. Additionally, tailored strategies for denture-wearers are crucial. These findings inform policies for enhanced oral health outcomes in similar contexts.

### Electronic supplementary material

Below is the link to the electronic supplementary material.

Supplementary Material 1

## Data Availability

All data generated or analyzed during this study are included in this published article & supporting material.
